# Serum proteomics mirrors the histopathological changes underlying different etiologies of primary mitral valve disease

**DOI:** 10.1042/BSR20253900

**Published:** 2026-01-14

**Authors:** Amaia Garcia-Peña, Jaime Ibarrola, Marina Segur, Adela Navarro, Alba Sádaba, Carolina Tiraplegui, Mattie Garaikoetxea, Ernesto Martín-Núñez, Amaya Fernández-Celis, Rafael Sádaba, Virginia Alvarez, Eva Jover, Natalia López-Andrés

**Affiliations:** 1 Navarrabiomed, Hospital Universitario de Navarra (HUN), Universidad Pública de Navarra (UPNA), IdiSNA, Pamplona, 31008, Spain; 2 CIBERCV, Carlos III Institute of Health, Madrid, Spain; 3 INI-CRCT, Nancy, France

**Keywords:** Barlow’s disease, calcific mitral valve disease, fibroelastic deficiency, myxomatous, rheumatic heart valve disease

## Abstract

Mitral valve disease (MVD) is the most common valvulopathy and a frequent cause of heart failure and death. However, data regarding the molecular basis are scarce. We aimed to thoroughly explore and compare the circulating, molecular, and histopathological profiles in the main subtypes of primary chronic MVD. In total, 300 patients with chronic primary MVD undergoing mitral valve (MV) replacement were enrolled and classified in the four main etiologic subtypes: calcific mitral valve disease (CMVD, *n* = 81), rheumatic disease (RHVD, *n* = 114), Barlow’s disease (BD, *n* = 70), and fibroelastic deficiency (FED, *n* = 35). Discovery studies were performed using Olink Proteomics^®^ technology in 80 serums from MVD patients (*n* = 20/etiology). Histopathologic study, ELISA, and zymography were performed on resected MVs to analyze extracellular matrix (ECM) composition and remodeling, inflammation, and calcification. Serum proteomics identified markers exclusively overexpressed in each MVD etiologic subtype. Further enrichment analyses revealed specific etiology-dependent pathways involving calcification, inflammation, or ECM remodeling. Such etiology-dependent differences were mirrored in the excised MVs both architecturally and compositionally. CMVD and RHVD valves had a marked ECM disorganization with increased collagen deposition and presence of nodular and diffuse calcification, respectively. Moreover, RHVD valves exhibited high levels of tumor necrosis factor (TNF)-α, transforming growth factor (TGF)-β, and matrix degradation enzymes. BD valves showed enhanced inflammatory markers and infiltrates. Moreover, BD and FED valves presented marked proteoglycans deposition. Our study identified circulating markers involved in calcification, inflammation, and ECM remodeling, which may be associated with specific MVD etiologies. The differences in serum markers seem to mirror histopathologic and molecular alterations in the MVs with potential applications into the clinic as diagnosis biomarkers. Identification of underlying molecular mechanisms of each etiology is essential to discover new specific therapeutic targets.

## Background

Mitral valve disease (MVD) is the most prevalent valvular heart disease; 1.8% of the general population in developed countries presents moderate or severe mitral valve (MV) dysfunction [1]. It can be caused by mitral regurgitation (MR), mitral stenosis (MS), or a combination of both. Primary MR is caused by an intrinsic abnormality of the leaflets, whereas secondary MR results from distortion of the MV apparatus due to left ventricle and/or left atrium remodeling [2].

The etiology of MVD is very varied and can be divided into inflammatory, degenerative, infective, structural, and congenital [3]. Myxomatous degeneration is the most common cause of primary MR in developed countries [4]. Classically, two different phenotypes of myxomatous degeneration have been described: diffuse myxomatous disease, also called Barlow’s disease (BD), and fibroelastic deficiency (FED) [5]. BD is characterized by increased leaflet thickness caused by proteoglycan accumulation [6]. FED is distinctive for diffuse chordal elongation in addition to chordal rupture [6]. Whether these subtypes are variants along a single spectrum or different diseases has long been debated [6,7].

Rheumatic heart valve disease (RHVD) is a sequel of acute rheumatic fever that triggers inflammatory valvular damage [8], being an important cause of cardiovascular death worldwide [9]. RHVD is the leading cause of MS [10].

Mitral annulus calcification (MAC) is a chronic degenerative process that predominantly affects the posterior fibrous base of the MV with an estimated prevalence of 8– 27% in the general adult population [11]. MAC commonly results in MV dysfunction, an entity recently termed calcific mitral valve disease (CMVD) [12,13]. Originally considered to be a result of chronic, age-related tissue degeneration leading to progressive calcium formation, MAC is now regarded as an active and complex molecular process [14].

Despite the high prevalence of MVD and the variety of different etiologies, data on the cellular and molecular mechanisms involved in the different etiologic subtypes are limited, and side-by-side comparison studies are lacking. Moreover, the research on biomarkers in MVD, both at the tissue or circulating level, is scarce. The discovery of etiology-specific biomarkers could potentially reveal the histopathological damages associated with MVD, help identify new targeted therapies, and be clinically relevant to the management of MVD patients. Since MVD etiologies are varied, we hypothesized that the specific changes in serum markers could mirror the histopathological modifications contributing to the understanding of MVD pathophysiology. Here, we classified 300 patients’ MVs, and a discovery study was performed by proteomics, molecular, and histological approaches in the main MVD etiological subtypes. We examined an unbiased proteomic analysis to identify new pathways involved in the development of each MVD etiology. We further analyzed the impact of these unbiased pathways and compared them with the main pathophysiological alterations described in heart valve diseases (inflammation, fibrosis, calcification).

## Methods

### Study population

The study consisted of 300 consecutive patients undergoing surgical MV replacement due to significant primary MVD in our institution according to current clinical practice guidelines [15]. Exclusion criteria were surgical repair and MVD caused by other etiologies (e.g., infectious endocarditis, ischemic MVD, or congenital). The presence of significant dysfunction or surgery of other valves was not considered an exclusion criterion, but these data were also collected and considered in the analyses.

The categorization of patients in the three main etiologic subtypes (BD, FED, CMVD, and RHVD) was based on the description of preoperative echocardiogram, according to current echocardiographic criteria [2,16,17] (Supplemental Table 1). BD valves are characterized by diffuse thickening and redundancy, typically affecting multiple segments of both leaflets and chordae, while FED refers to focal segmental pathology with thin leaflets. CMVD was defined by the dysfunction caused by the presence of MAC, observed in echocardiography as an echodense structure with acoustic shadowing involving the posterior mitral annulus and frequently extending to the posterior leaflet. Rheumatic valves were characterized by commissural fusion, leaflet thickening with or without calcification, and restricted leaflet motion, which causes the typical diastolic doming of the anterior leaflet in long axis or ‘fish-mouth’ appearance in the short axis, as well as chordal leaflet thickening. All patients underwent transthoracic echocardiography performed by cardiologists experienced in cardiac imaging. Intraoperative description by the cardiac surgeon and revision of the recorded images by the first author were included in inconclusive reports.

Peripheral blood samples were extracted within 24 h prior to the surgery for routine analyses. Control valves (*n* = 18, 36% women, 58 ± 14 years old) were obtained from patients undergoing cardiac transplantation due to dilated or ischemic cardiomyopathy, or from organ donors without cardiac disease whose hearts could not be used as grafts, after demonstration of the absence of relevant alterations in histological analysis.

### Targeted proteomics in serum samples

High-throughput real-time proteomics was performed for biomarkers identification using the Proximity Extension Assay (PEA) technology (Olink® Target 96 Cardiovascular III panel, [Olink® Bioscience, Uppsala, Sweden]). Briefly, 80 serums (*N* = 20/each MVD etiology) were assayed in a panel of 92 cardiovascular-related proteins for target discovery at Cobiomic Bioscience (Córdoba, Spain; https://cobiomicbioscience.com/). For each etiologic subtype, patients were matched by clinical parameters (sex, age, treatments, echocardiographic parameters). The PEA technology has been described in detail elsewhere (https://www.olink.com/). The protein expression values are presented in arbitrary units (NPX values; https://olink.com/faq/what-is-npx/) in the log2 scale. Intra- and inter-assay coefficients of variations, detection limits, and biological information for each protein are reported on the manufacturer’s website (https://www.olink.com/). Olink technology uses 92-target panels to perform high-multiplex protein analysis for biomarker discovery in specific disease areas or biological processes. Cardiovascular panel III was chosen because it is focused on inflammation, extracellular matrix (ECM) proteins, and coagulation.

Heat maps were elaborated to evidence specific differential expression patterns across the four etiologies assessed. Venn diagrams were drawn to find out both targets exclusively up-regulated within each etiology and the existence of shared commonalities across the different etiologies. All the enhanced targets evidenced by the heatmap were included. Moreover, Multivariate Analysis of Variance was performed to reveal those targets significantly or tendentiously up-regulated. Finally, functional enrichment annotations were conducted using Enrich R and the potential degree and evidence of interaction among the targets was assessed by drawing STRING interactomes. The list of targets found significantly or tendentiously up-regulated were input in both cases. Only the terms with a *P* value < 0.05 were considered, and the information was plotted by the expression odds ratio (OR). Across the four etiologies, selected markers, significantly or tendentiously different, were further validated by ELISA in serum from the whole cohort.

### Histology

Human MVs were fixed in 4%-buffered formol for 24 h. Histological determinations were performed in 5 μm-thick sections that were stained with Movat Pentachrome, Alcian blue/Sirius red, orcein, and alizarin red following the manufacturer’s instructions (Sigma).

The immunochemistry was performed following the protocol of Leica BOND-Polymer Refine Detection automatic immunostainer (Leica). All solutions were filled into the bottle-Bond Open Container (Leica) and registered on computer using the Leica Biosystem program. The immunostaining program protocol includes Fixative solution, Bond wash solution, Blocking solution (animal serum to prevent nonspecific antibody binding, suspended in Tris-buffered saline (TBS) and preserved with 0.1% ProClin™ 950, an antimicrobial agent) and incubated with the primary antibody for CD68 (Abcam), CD45 (Santa Cruz Biotechnology), Periostin (Santa Cruz Biotechnology), α-SMA (Santa Cruz Biotechnology) and vimentin (Santa Cruz Biotechnology). After primary antibody incubation, slides were incubated with postprimary poly-HRP-IgG. The signal was revealed by using DAB substrate. As negative controls, samples followed the same procedure described above but in the absence of primary antibodies were used. For each immunochemistry and staining, serial sections were done. In the figures, the most representative images are shown. We have analyzed at least ten different MVs from each etiologic subtype. For the immunohistochemistry quantification, five different areas from ten different valves were analyzed using ImageJ software.

## ELISA

Single ELISA kits were used to measure target-specific proteins; Glycoprotein VI (GP6) (dilution 1/5), ST2 (dilution 1/200), noncanonical Notch ligand 1 (DLK1) (dilution 1/200), epidermal growth factor receptor (EGFR) (dilution 1/200), peptidoglycan recognition protein 1 (PGLYRP1) (dilution 1/200), growth differentiation factor 15 (GDF15) (dilution 1/10), trefoil factor 3 (TFF3) (dilution 1/200), osteopontin (OPN) (dilution 1/200), Collagen type I, Transforming growth factor (TGF)-β, aggrecan, lumican, syndecan-1, decorin, metalloproteinase (MMP)-1, tissue MMP inhibitor (TIMP)-1, MMP-2, TIMP-2, MMP-9, C-C motif chemokine 5 (Rantes), interleukin (IL)-1β, IL-6, Tumor necrosis factor (TNF)-α, bone morphogenetic protein (BMP)-2 and BMP-4, osteocalcin, receptor activator of nuclear factor kappa beta ligand (RANK-L) and periostin were measured in valve protein extracts (at 5 µg) by ELISA according to the manufacturer’s instructions (R&D Systems). The specific serum dilution for each ELISA was previously pretested and verified to ensure that it was optimal for the assay.

### Gelatin zymography

Extracellular MMP activity was assessed in human MV lysates by gelatin zymography. Gelatin gels were prepared using 10% SDS-polyacrylamide gels containing 0.3% gelatin. Equal volumes (30 µl) of supernatant samples were diluted into 2X tris-glycine SDS sample buffer (BioRad) and electrophoretically separated under nonreducing conditions. Gels were washed three times for 15 minutes with a solution of 2.5% Triton X-100. After washing, gels were incubated overnight at 37°C in incubation buffer (1 M TRIS adjusted to pH 7.5, 1 M CaCl_2_, 5 M NaCl, 0.05% Tween) to promote gelatinase activity. Following overnight incubation, gels were stained with 0.25% Coomassie Blue for 30 minutes and de-stained with 10% acetic acid and 50% methanol in water. Fold changes in band densitometries are expressed in arbitrary units (AU).

### Real-time reverse transcription PCR

Total RNA was isolated according to a standardized phenol-chloroform protocol, using Qiazol reagent and miRNeasy mini Kit (217004, QIAGEN, Germany), and reverse-transcribed into single-stranded cDNA, using an iScript Advanced cDNA Synthesis Kit (Bio-Rad). Downstream qPCR amplification of first-strand cDNA was performed using iQ SYBR Green Supermix (Bio-Rad) in a CFX Connect Real-Time PCR System (Bio-Rad). The relative expression of each selected gene product was calculated using the 2−ΔΔCt method. All reactions were performed in technical triplicates. The following primer was used; ACTA2 (Forward: ACTGCCTTGGTGTGTGACAATGG; Reverse: TGGTGCCAGATCTTTTCCATG) and VIM (Forward: AGGCGAGGAGAGCAGGATTT; Reverse: AGTGGGTATCAACCAGAGGGA).

### Statistical analyses

Continuous data are expressed as mean ± standard deviation (SD) and categorical data as frequency (percentage). Clinical data were analyzed with IBM SPSS Statistics 25.0.0.0 software. Normal distribution of data was evaluated by the Kolmogorov-Smirnov test. One-way ANOVA was used in continuous variables and Pearson’s χ2 test in categorical variables to assess statistical differences between groups. Molecular data were analyzed using GraphPad Software Inc, using one-way ANOVA or Kruskal–Wallis test followed by Dunnett’s test or Mann–Whitney U tests to assess differences between each etiologic group and control valves. In each analysis, the critical significance level was set to a *P* value of < 0.05.

Multivariate analysis of variance (MANOVA) was conducted using SPSS software to analyze, at once, the existing differences found by high-throughput proteome analysis (i.e., 92 proteins) across the four etiology groups. Levene test was used to assess the data’s homoscedasticity; HSD Tukey or T3 Dunnet were the post hoc tests used when assuming or not equal variances, respectively. Those proteins significantly up-regulated in one group compared with the others were listed for ulterior functional annotation enrichments and additional validations by ELISA. Moreover, markers having a *P*<0.150 were also considered to represent a ‘tendency’ toward significance to be included in the enrichment analyses and further considered for validation in independent samples.

## Results

### Clinical characteristics

Supplemental Table 1 shows clinical characteristics of the patients enrolled in this study. RHVD (38%) was the most numerous group of patients. CMVD and FED patients were the oldest (74.7 ± 6.0 and 73.7 ± 8.6, respectively), although the CMVD group had the highest proportion of concomitant cardiovascular risk factors including the highest rate of coronary heart disease (34.6%). There was a female predominance in CMVD and RHVD, while in BD and FED, male sex was preponderant, similar to other reports [8,11,18]. As expected, atrial fibrillation was more prevalent in RHVD patients. Accordingly, at baseline, the use of beta-blockers, digoxin, and vitamin K antagonists was higher in this group (Supplemental Table 3). Laboratory parameters are found in Supplemental Table 4.

### Proteomic profile

**Figure 1:**
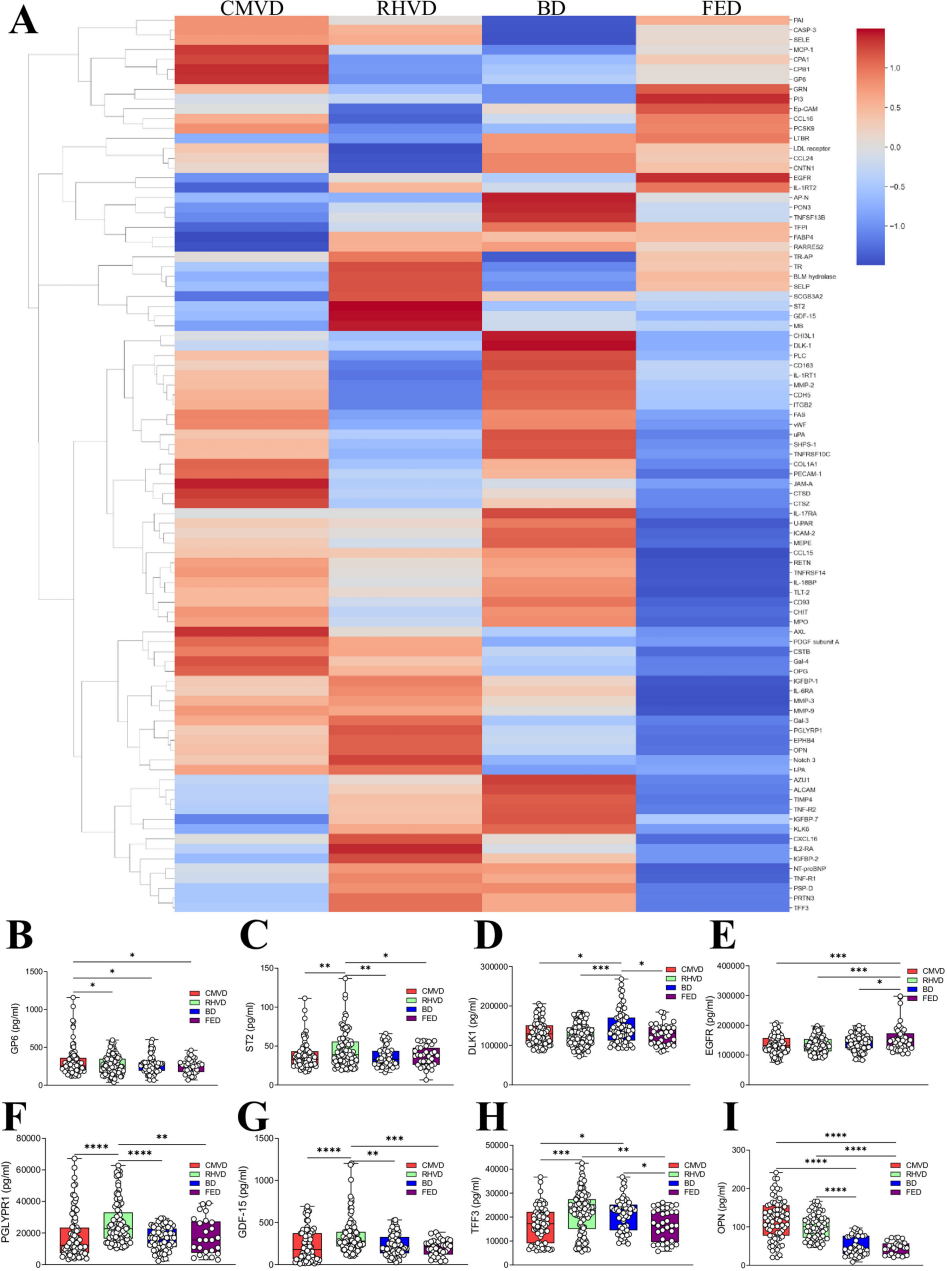
Serum proteome profiling in the main forms of MVD. **A,** Heatmap plotting the NPX data generated by OLINK technology and analysis in the main forms of MVD including CMVD, RHVD, BD, and FED. **B-I**, Validation analyses for selected targets significantly and exclusively up-regulated in each etiology of the whole cohort of primary chronic MVD measured in serum. *N* = 300 MVD patients (*N* = 81 for CMVD, *N* = 114 for RHVD, *N* = 70 for BD, *N* = 35 for FED). One-way ANOVA or Kruskal–Wallis test followed by Dunnett’s or Mann–Whitney U test were performed. BD, Barlow’s disease; CMVD, calcific degenerative mitral valve disease; FED, fibroelastic deficiency; RHVD, mitral valve disease. *P<0.05; **P<0.01; ***P<0.001; ****P<0.0001.

In order to evidence the existing differences between the 4 MVD etiologies assessed in this manuscript, we performed a high-throughput proteome analysis (OLINK®) looking at the expression of 92 proteins. We performed a bulk analysis of all the targets’ expression across the four groups to identify these specifically up-regulated within each etiology. Multivariate analysis of the Olink Proteomics® panel data showed that six targets were exclusively up-regulated in CMVD, seven in RHVD, ten in BD, and three in FED (Supplemental Figure 1A-B). Heatmap and Venn diagram (Figure 1 and Supplemental figure 1A) as well as with the up-regulated targets, significantly or tendentiously significant, from the high-throughput OLINK® proteome analysis. These interactomes show both the interaction among the identified targets (colored lines) as well as the expression level of each one (light-to-dark red ring meaning a lower-to-higher expression). Regarding the color of the lines: black lines refer to target co-expression; pink lines, mean known interactions experimentally determined; light green, mean interactions evidenced by text mining; light blue represent known interactions from curated databases. Moreover, the pathways potentially regulated by these enhanced targets within each MVD etiology were functionally annotated using Enrich R to highlight clinically relevant pathogenic mechanisms in CMVD, RHVD, BD, or FED.

Considering the results of this discovery approach, the most differentially expressed molecules were further validated in the whole cohort. Thus, glycoprotein VI (GP6) serum levels were increased in CMVD as compared with the other etiologies (Figure 1B). Serum ST2 was elevated in RHVD (Figure 1C). The expression of serum delta-like noncanonical Notch ligand 1 (DLK1) was enhanced in BD (Figure 1D), whereas epidermal growth factor receptor (EGFR) levels were augmented in FED patients (Figure 1E). Complementary analyses were performed on potentially etiology-specific targets showing statistical trends by high-throughput real-time proteomics. As suggested by our OLINK results (Supplemental Figure 1B, ‘Targets exclusively up-regulated’), peptidoglycan recognition protein 1 (PGLYRP1) and growth differentiation factor 15 (GDF15) levels were increased in RHVD (Figure 1F–G). Additionally, trefoil factor 3 (TFF3) and osteopontin (OPN) levels were higher in CMVD and RHVD as compared with BD and FED (Figure 1H–I).

Further enrichment analysis considering etiological-specific targets was done. Pathway analysis showed that in CMVD patients, there was a higher presence of osteoblast signaling, inflammation, and collagen type I synthesis pathways (Supplemental Figure 1C-D), compatible with active osteoinflammatory cues characteristic of ectopic calcification. In RHVD, interaction analysis showed significant enrichment in pathways associated with rheumatoid arthritis genes and inflammation mediators (Supplemental Figure 1E-F), suggesting a strong and predominant inflammatory response accompanied by abundant fibro-calcific deposits found in RHVD specimens. Pathway enrichment analysis in BD mainly identified processes related to macrophages and inflammation (Supplemental Figure 1G-H). Finally, the biological pathways significantly enriched in FED were vaguer and involved inflammation EGFR pathway and actin cytoskeleton (Supplemental Figure 2I-J).

### Different patterns of ECM components in MVs from each etiologic subtype

**Figure 2:**
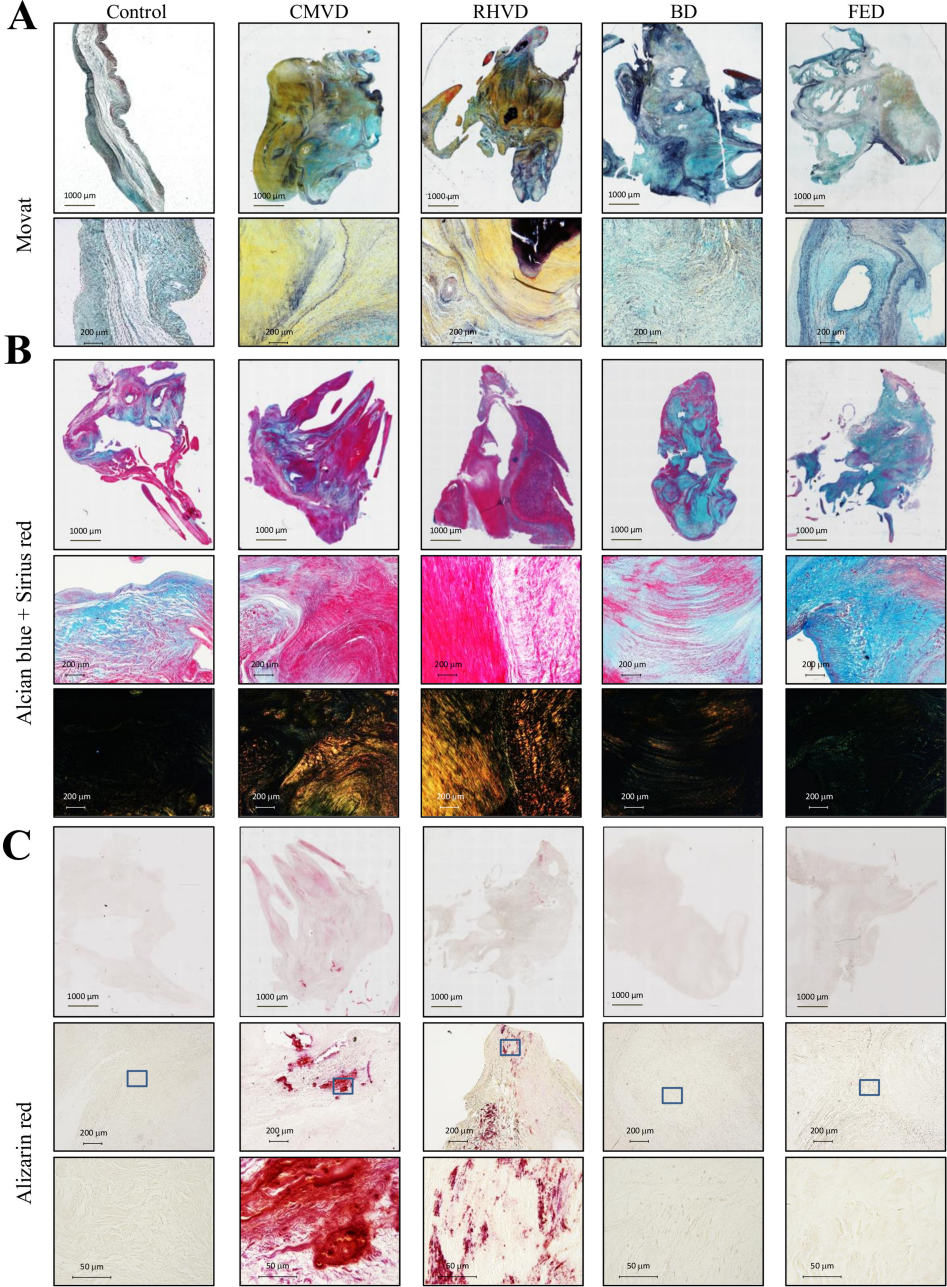
Histopathological characterization of the extracellular matrix architecture and remodeling processes in each MVD etiology. **A**, Representative microphotographs of the whole piece of tissue stained with Movat (top panel) and at low magnification (50X, bottom panel). Blue, glycosaminoglycans; light yellow, collagen; dark blue/purple, elastic fibers; bright yellow, calcification/bone; bright red, cartilage/inmature bone. **B**, Representative microphotographs of Alcian blue (proteoglycan content) and Sirius Red (collagen content) staining. Bright field captures are displayed for the whole piece of tissue on the top panel. Pictures at high magnification (200X) imaged in bright field or polarized light are displayed in the medial or bottom panels, respectively. Polarized light images show in red-yellow thick collagen fibers and in green thin collagen fibers. **C**, Representative microphotographs of the whole piece of tissue stained with Alizarin red (calcification) (top panel), at low magnification (50X, middle panel) or high magnification (400X, bottom panel). BD, Barlow’s disease; CMVD, calcific degenerative mitral valve disease; FED, fibroelastic deficiency; RHVD, mitral valve disease.

We next ought to analyze whether the etiology-enriched mechanisms evidenced upon serum and tissue profiling could reflect architectural and compositional differences in MV. For this purpose, MV tissues were histologically analyzed (Figure 2A–C). Of note, a control group of MV tissues was added. Movat pentachrome staining revealed structural and composition differences between control and diseased MVs, with such differences strongly evident among the four MVD etiologies. While control valves were composed of loose parallelly arranged proteoglycan fibers (light blue staining), valves with myxomatous degeneration (BD and FED) showed increased proteoglycans deposition (Figure 2A–B). BD valves showed spongiosa layer expansion due to a diffuse framework of proteoglycans accumulation, whereas FED valves exhibited a more condensed proteoglycans deposition (Figure 2A–B). CMVD and RHVD valves revealed marked ECM disorganization with extensive and disrupted collagen deposition (yellow staining), with intercalating fragmented and disarrayed elastin fibers as well as an increased VIC population together with a high number of active VICs (Figure 2A–B). To demonstrate the increase in VIC population, markers of activated VICs (α-SMA and vimentin) were measured. As shown in representative immunostaining, the expression of VIC markers was increased in CMVD as compared with controls (Supplemental Figure 2A-B). Quantification of α-SMA and vimentin mRNA levels (ACTA2 and VIM) confirmed that both were significantly increased in the CMVD group (Supplemental Figure 2B-C).

Figure 2B displays double alcian blue (proteoglycans)/Sirius red (collagen) staining and posterior analysis under polarized light to illustrate the relative abundance of thick fibers (red/yellow) and thin fibers (green) of collagen. Control valves showed loose parallel collagen fibers, predominantly thin, while in CMVD, there was dense linear and nodular deposition of collagen thick fibers in the fibrosa layer and diffuse deposition of thin collagen and intercalated diffuse glycosaminoglycans in the spongiosa layer. Interestingly, RHVD revealed extensive collagen fibrosis, mainly composed of thick collagen and low or even absent amounts of GAGs. In BD valves, the pattern was different. BD valves were composed mainly of proteoglycans and similar amounts of thick-thin collagen fibers. In FED valves, there was deposition of thin fibers.

Figure 2C shows that Alizarin red staining was markedly increased in CMVD and RHVD. CMVD valves showed characteristic calcific nodules, whereas rheumatic valves presented more diffuse calcification. BD and FED leaflets did not reveal significant calcification.

### Fibrosis and ECM remodeling in primary MVD

**Figure 3:**
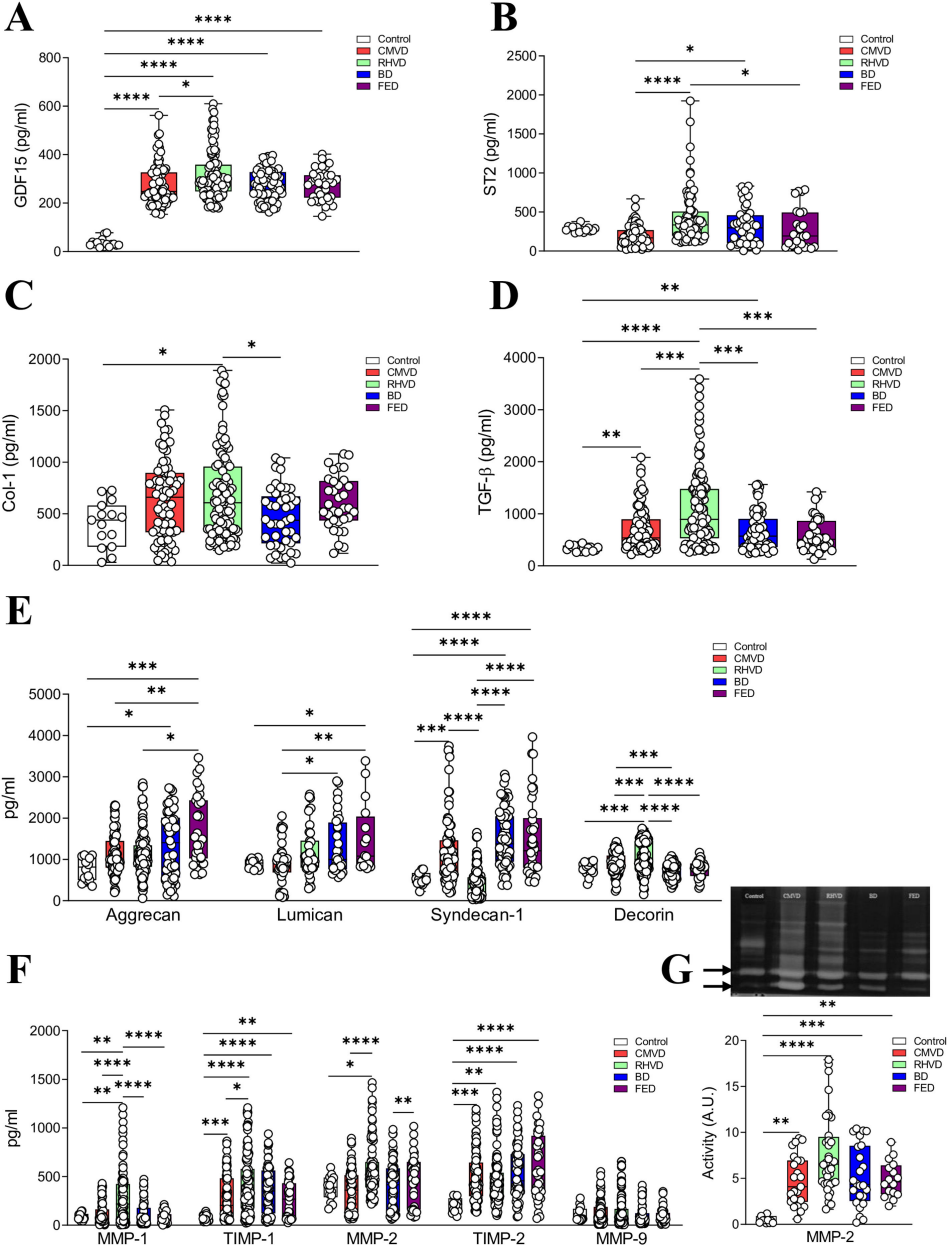
Etiological-dependent characterization of markers for extracellular matrix remodeling and fibrosis. MV tissue homogenates of control, CMVD, RHVD, BD and FED groups were assayed. Quantification of GDF-15 (**A**) and ST2 (**B**) in MV from the whole clinical cohort. **C-D,** Content of content of collagen type-I (Col-1) and TGF-β (transforming growth factor β) measured by ELISA. **E**, Content of proteoglycans (aggrecan, lumican, syndecan-1 and decorin) in MVs measured by ELISA. **F**, Quantification of matrix metalloproteinase (MMP)-1, tissue inhibitor of matrix metalloproteinase (TIMP)-1, MMP-2, TIMP-2, MMP-9 in MVs measured by ELISA. **G**, MMP-2 activity in MVs measured by zymography. A representative image of a Comassie blue-stained zymography per each etiologic class has been displayed on the top of the graph. *N* = 300 MVD valves were analyzed (*N* = 81 for CMVD, *N* = 114 for RHVD, *N* = 70 for BD, *N* = 35 for FED). One-way ANOVA or Kruskal–Wallis test followed by Dunnett’s or Mann–Whitney U test were performed. BD, Barlow’s disease; CMVD, calcific degenerative mitral valve disease; FED, fibroelastic deficiency; RHVD, mitral valve disease. *P<0.05; **P<0.01; ***P<0.001; ****P<0.0001.

We next integrated the results from the proteomic discovery phase, the enrichment analysis (in serum), and the histological findings (in tissue sections). For this purpose, quantitative analysis of the main proteins identified in the discovery analysis, or involved in the pathophysiological processes described by the pathway enrichment analysis, was quantified in equal amounts of MV tissue homogenates from the different etiological subtypes. To start, the quantification of ECM proteins involved in MVD development was performed. First, GDF15 and ST2 (obtained from the proteomic analysis, Figure 1 and Supplemental Figure 1) were measured in MV tissue. Quantitative results confirmed that both GDF-15 and ST2 levels were enhanced in RHVD (Figure 3A–B). In agreement with our pathway enrichment analysis as well as with the histopathological observations, CMVD and RHVD valves presented increased amounts of collagen type I, with RHVD having the highest levels (Figure 3C). The levels of the master regulator of fibrosis, TGF-β, were elevated in RHVD (Figure 3D). This finding is consistent with Figure 3C, where collagen type 1 increases only in RHVD.

In line with the histological studies, proteoglycan analyses revealed higher levels of aggrecan (Figure 3E) in FED valves. In parallel, BD and FED valves showed increased expression of the small leucine-rich proteoglycan lumican (Figure 3E). Moreover, the cell sulfate proteoglycan syndecan-1 presented a different pattern of expression, being significantly increased in CMVD, BD, and FED as compared with controls and RHVD valves (Figure 3E). The enhanced accumulation of syndecan-1 might in part explain the presence of diffuse glycosaminoglycans in the spongiosa layer of CMVD as compared with the fewer or absent in RHVD. Decorin concentrations were only increased in RHVD valves (Figure 3E) in line with the abundant accumulation of thick cross-linked collagen type-1 found in RHVD samples.

Quantification of the principal ECM degradation enzymes (MMPs) and their inhibitors (TIMPs) showed increased expression of MMP-1 and TIMP-1 in RHVD relative to controls and the other etiologic subtypes (Figure 3F). MMP-2 expression was enhanced in RHVD as compared with Control and CMVD valves, and in FED vs. BD valves (Figure 3F). MMP-9 levels were similar between controls and diseased MVs (Figure 3F). Protein levels of TIMP-2 were significantly increased in all etiologic subtypes as compared with controls (Figure 3F). Moreover, MMP-2 enzymatic activity was significantly augmented in all types of MVD, being elevated in RHVD (Figure 3G).

### Inflammatory markers in MVD

**Figure 4:**
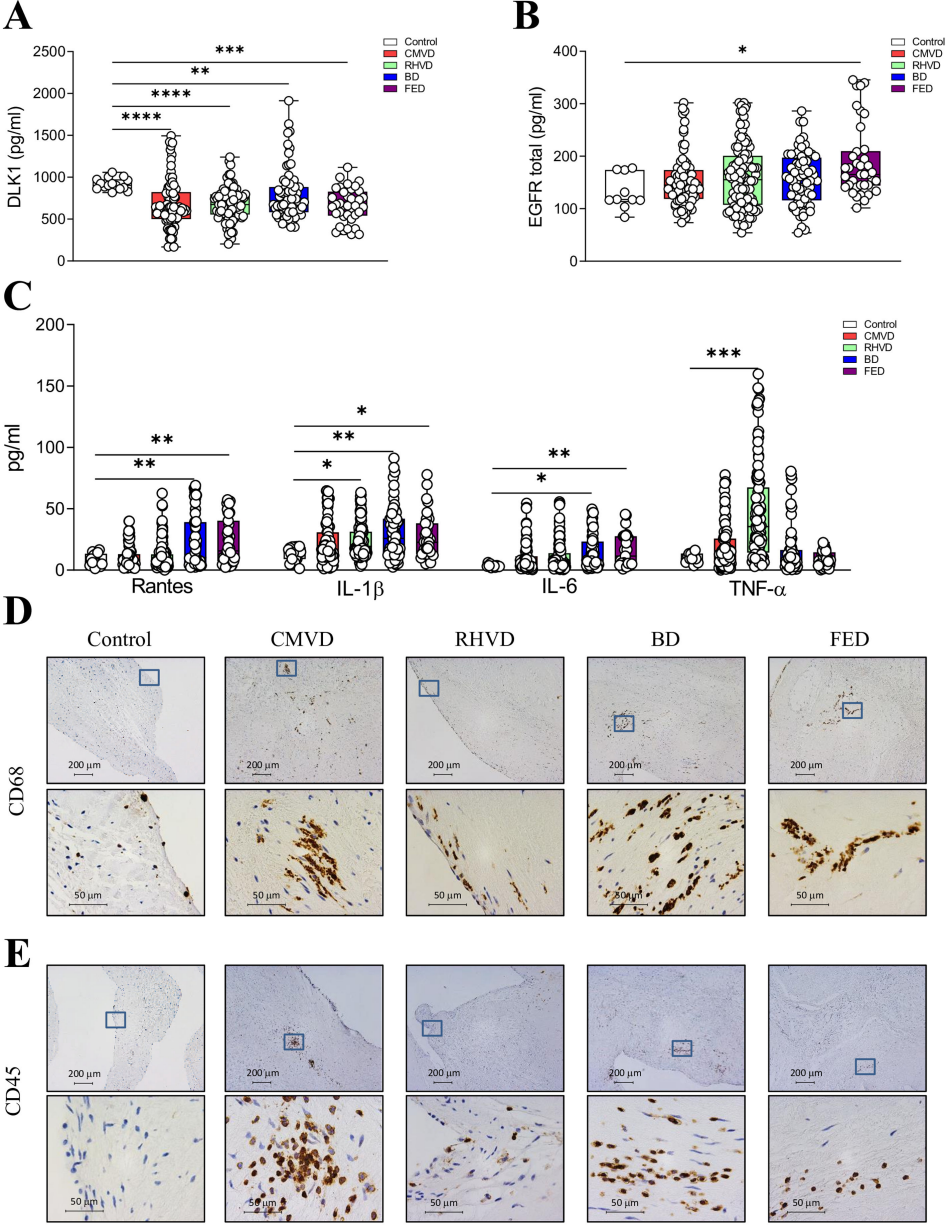
Etiological-dependent profiles of inflammatory markers. MV tissue homogenates of control, CMVD, RHVD, BD and FED groups were assayed. Quantification of DLK-1 (**A**) and EGFR (**B**) in MV from the whole clinical cohort. **C**, Quantification of inflammatory cytokines (Rantes, interleukin (IL)-1β, IL-6 and tumor necrosis factor (TNF)-α) in MV measured by ELISA. **D**, Immunohistological characterization of macrophages infiltrates (CD68) at low (50X) or high (400X) magnification (top and bottom panels, respectively). **E**, Immunohistological characterization of leukocyte infiltrates (CD45) inflammatory infiltrates at low (50X) or high (400X) magnification (top and bottom panels, respectively). *N* = 300 MVD valves were analyzed (*N* = 81 for CMVD, *N* = 114 for RHVD, *N* = 70 for BD, *N* = 35 for FED). One-way ANOVA or Kruskal–Wallis test followed by Dunnett’s or Mann–Whitney U test were performed. BD, Barlow’s disease; CMVD, calcific degenerative mitral valve disease; FED, fibroelastic deficiency; RHVD, mitral valve disease. *P<0.05; **P<0.01; ***P<0.001; ****P<0.0001.

Some of the inflammation markers obtained from the proteomic analysis were measured in MV tissues. DLK1 protein expression diminished in all subtypes when compared with control valves, although higher levels in diseased valves were found in BD (Figure 4A). EGFR was higher in FED valves (Figure 4B). Quantification of classical inflammatory markers such as Rantes, interleukin (IL)-1β, IL-6, and TNF-α demonstrated significant differences among MVD subtypes (Figure 4C). BD and FED valves were the only subtypes that presented significantly higher levels of Rantes and IL-6 vs. controls in agreement with macrophage-related enrichment shown in Supplemental Figure 1G-J. RHVD valves showed dramatically higher TNF-α levels than any other subtype, and also higher levels of IL-1β. Conversely, CMVD valves did not present significant differences to controls regarding the inflammatory profile.

Presence of inflammatory cells within the MVs was further characterized by immunostaining for CD68 (macrophages) and CD45 (leukocytes) (Figure 4D–E). Quantification of CD68 and CD45 showed a significant increment of inflammatory cells in all etiologies compared with the control group (Supplemental Figure 3A-B). In CMVD and BD, inflammatory infiltrates were present in clusters within the leaflet. FED leaflets also showed inflammatory aggregates, mainly consisting of macrophages. RHVD valves presented scarce inflammatory cell infiltration (Figure 4D). Interestingly, the expression of CD45 was higher in CMVD and BD valves (Figure 4E).

### Differences in calcification patterns and osteogenic protein expression in MVD

**Figure 5:**
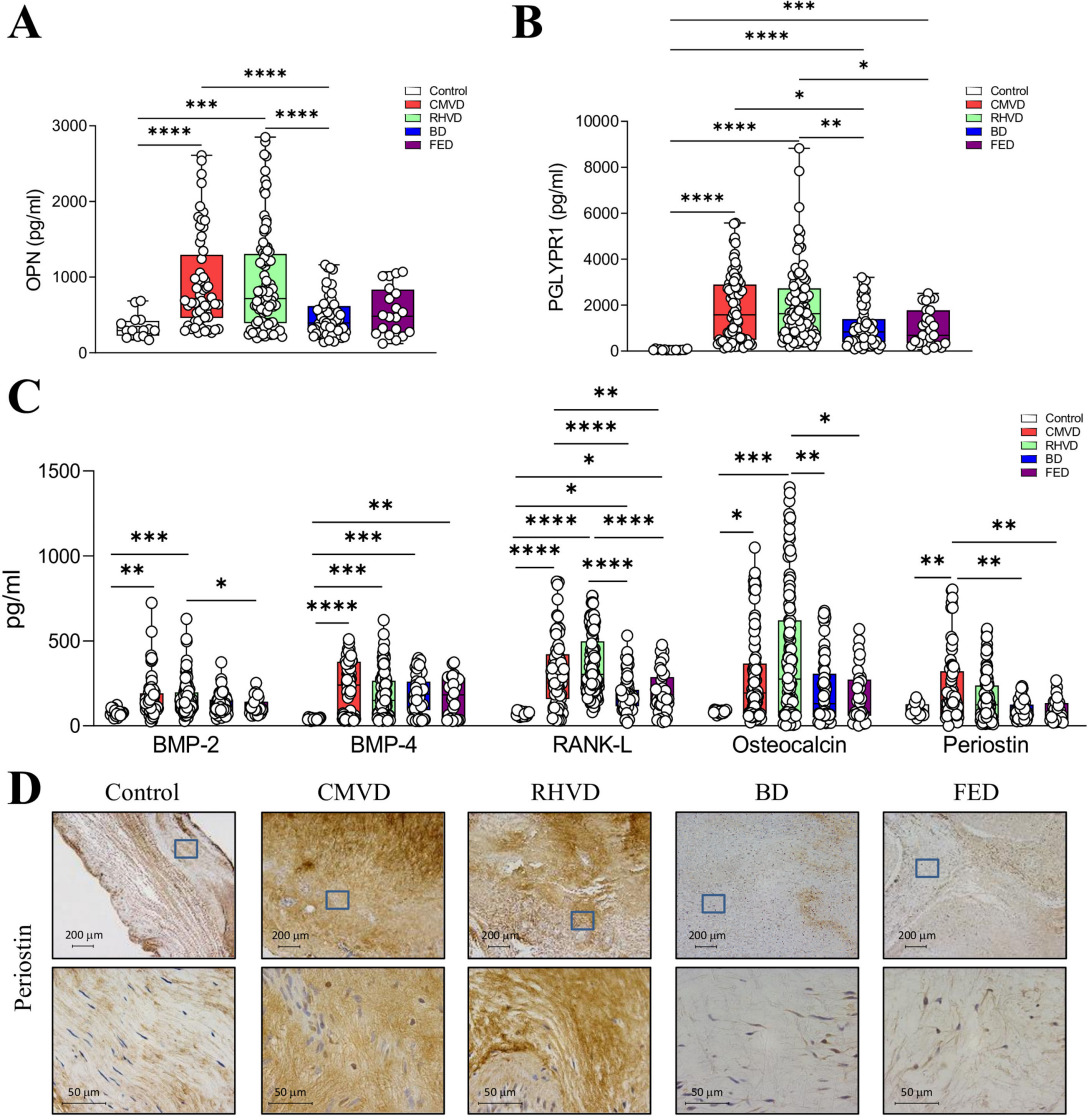
Etiological-dependent profiles of osteogenic markers. MV tissue homogenates of control, CMVD, RHVD, BD and FED groups were assayed. Quantification of Osteopontin (**A**) and PGLRYRP1 (**B**) in MV from the whole clinical cohort. **C**, Quantification of osteogenic markers (bone morphogenic proteins (BMP)-2 and BMP-4, RANK-L, osteocalcin, periostin) measured in MVs by ELISA. **C**, Immunohistological characterization of periostin expression at low (50X) or high (400X) magnification (top and bottom panels, respectively). *N* = 300 MVD valves were analyzed (*N* = 81 for CMVD, *N* = 114 for RHVD, *N* = 70 for BD, *N* = 35 for FED). One-way ANOVA or Kruskal–Wallis test followed by Dunnett’s or Mann–Whitney U test were performed. BD, Barlow’s disease; CMVD, calcific degenerative mitral valve disease; FED, fibroelastic deficiency; RHVD, mitral valve disease. **P<0.01; ***P<0.001; ****P<0.0001.

The expression of two markers related to calcification processes, OPN and PGLYRP1 (obtained from the proteomics analysis), was quantified in MV tissue homogenate. The expression of OPN levels was enhanced in CMVD and RHVD as compared with control and BD valves (Figure 5A). Similarly, PGLYRP1 was higher in CMVD and RHVD as compared with control and BD valves (Figure 5B), correlating with the previous results in serum (Figure 1F–1I). To investigate the role of bone metabolism in each etiologic subtype, we quantified the expression of the classical bone proteins (e.g. BMP-2 and -4, RANK-L, osteocalcin, periostin) (Figure 5C). When comparing the four MVD etiologies, CMVD and RHVD valves presented the highest levels for most of the calcification markers including BMP-2, RANKL, and osteocalcin. More precisely, BMP-2 and OCN levels were enhanced in RHVD valves, and BMP-4 was increased in CMVD valves, whereas RANKL expression was elevated in both CMVD and RHVD valves (Figure 5C). These results correlate with Figure 2C where calcification staining is only present in CMVD and RHVD. Periostin levels were significantly higher in CMVD valves than in control, BD, and FED valves (Figure 5C–D).

## Discussion

To our knowledge, this is the most thorough comparative analysis on MVD. Using a discovery and translational approach, the present study demonstrated that each etiologic subtype presents a distinctive circulating, histologic, and molecular profile of the pathophysiologic mechanisms involved in MVD development (Central Illustration). We used an Olink proteomic panel to address the discovery of possible new specific targets that may explain differences among MVD etiologic subtypes. Most of the Olink proteomic targets were validated in serum and MV tissue from our MVP patients. Our data showed targets differentially expressed in serum from MVD patients, depending on the etiologic subtype. Enrichment analysis revealed that there was indeed a representation of different biological processes and pathways between MVD subtypes. Thus, in CMVD, there was a greater presence of osteoblast signaling, inflammatory pathways, or collagen metabolism, while in those of RHVD, there was a greater abundance of rheumatoid arthritis and fibro-inflammatory pathways. Concerning BD, enrichment analysis showed an over-representation of macrophage and inflammatory pathways, whereas in FED, there was higher abundance of inflammation, EGFR pathway, or actin cytoskeleton remodeling. Thus, inflammation emerges as a common pathway for all MVD subtypes, whereas each etiologic subtype exhibits differential characteristics.

### ECM architecture

The homeostasis of the ECM is fundamental to the biomechanical properties of the MV. Hence, the quantity, quality, and organization of the ECM components (particularly collagen, elastin, and proteoglycans) determines the correct adaptability, pliability, and overall long-term durability of the MV [19,20]. Similar to what has been previously described [21], we observed marked disarrayed disposition of fibrillar collagen in RHVD valves, which presumably contributes to valvular stiffening and dysfunction. MMP-2 activity was increased in both RHVD and CMVD valves. Of note, MMP-2 is also able to activate latent TGF-β [22] that was dramatically increased in RHVD valves as compared with the other MVD subtypes. This data is in accordance with other studies showing that high TGF-β expression positively correlated with the proliferation of VICs, fibrosis, inflammation, and calcification in RHVD valves [23,24]. All these data correlated with our proteomics finding where GDF15, a divergent member of the TGF-β superfamily and ST-2, a marker of cardiac fibrosis, were elevated in RHVD. Both GDF-15 and ST2 could induce collagen accumulation [25,26]. Of note, studies have demonstrated a positive correlation between GDF-15 and ST2, suggesting that they could be influenced by similar pathways [27]. So, it is plausible to hypothesize that serum increase in GDF-15 and ST2 could be related to enhanced collagen accumulation in MV, specifically in RHVD.

### Proteoglycans

Decorin and biglycan are found to be expressed predominantly in the flow-exposed atrialis layer of the healthy MV [19]. Decorin also participates in collagen fibril formation and stabilization [19]. Although its role in CMVD has not been explored, in calcified aortic valves, decorin has been found to be significantly more abundant in the calcific prenodule and surrounding region, suggesting a role in the earlier stages of valve calcification [28]. This could explain our results, where decorin is not increased in CMVD, reasonably due to our MV from CMVD being in an advanced stage of calcification. Previous studies showed that decorin can bind TGF-β with high affinity, thereby attenuating the profibrotic effect of TGF-β [29]. However, a proinflammatory role of decorin has been described, resulting in increased TNF-α synthesis [30]. Moreover, decorin is sensitive to MMP-2, which can digest core proteins and release cytokines such as TGF-β or TNF-α previously bound to decorin [30]. Interestingly, we demonstrated significantly higher levels of decorin, TGF-β, TNF-α, and MMPs levels and activity in RHVD valves, suggesting a role of this pathway in the development of this valvulopathy.

Syndecan-1 was significantly elevated in all subtypes except RHVD. The role of syndecan-1 in cardiac pathophysiology is complex and depends on the context and on the mechanism of injury [31]. Targeted deletion of syndecan-1 resulted in increased inflammation, increased activity of MMP-2 and -9, and deficient collagen maturation and organization [32]. Increased MMP activity is, in turn, associated with latent TGF-β activation [23]. However, a profibrotic role of syndecan-1 has also been described with increased cardiac expression of collagens [33]. That may be consistent with our findings: higher levels of syndecan-1 in myxomatous valves may play a profibrotic role, whereas in the predominantly inflammatory RHVD valves, the lowered levels of syndecan-1 may contribute to further valvular degradation and ECM remodeling.

### Inflammation

Inflammation is a key mediator of pathological valve remodeling and was found as a common pathway in all four MVD etiologies, by Olink proteomic, serum, and MV tissue analysis. Furthermore, our enrichment analyses on serum samples and ulterior molecular and histological assessment of the MV leaflets suggest that specific inflammatory cells and mechanisms differentially contribute to the MVD, owing to different inflammatory cues that may lead to the development of the etiology-dependent phenotypes described in this paper. Myxomatous valves presented significantly higher levels of proinflammatory cytokines. Recently, a correlation between serum levels of inflammatory markers and the severity of myxomatous MVD has been reported in dogs [34]. This study correlated with our results where RANTES, IL-1β, and IL-6 are increased significantly in BD and FED subtypes. This correlated also with the proteomic analysis, where EGFR (previously associated with inflammation in aortic stenosis [35]) was increased in FED. Interestingly, DLK-1 (which acts as an inhibitor of inflammation by suppressing NF-κB activity) was decreased in all MVD valves [36]. Surprisingly, RHVD valves only showed a significant increase in TNF-α compared with controls. In RHVD, *in vitro* studies have demonstrated that TNF-α exhibits a high chemotactic potential for inflammatory cells [8]. Systemic levels of this cytokine were associated with severity of RHVD and co-regulated expression of IL-6 and TNF-α was associated with more severe valvular dysfunction [8].

### Calcification

Previous studies demonstrated that valve calcification is not a passive process but rather a complex and regulated process associated with the expression of osteogenic markers that eventually leads to bone formation [37]. However, these studies were mainly based on aortic stenosis. In MAC, the calcification progression into the leaflet starts in the annulus and contributes to the calcification phenotype [38] but the mechanism is unknown. Of importance, our discovery analysis revealed an enrichment in osteoblast signaling in RHVD. To our knowledge, this is the first work that specifically assesses the molecules involved in calcification in CMVD caused by senile degeneration. Calcification was predominant in CMVD and RHVD by histological and molecular analysis, although our results suggest differences in the molecules involved in calcification between both subtypes. Of interest, these data were in accordance with serum osteopontin and PGLYRP1, which were enhanced in both CMVD and RHVD, being both molecules related to calcification in other valvular diseases or in atherosclerosis [39,40]. Further studies are warranted to analyze more in depth the precise role of each of the mediators contributing to osteogenesis in the MV.

**Figure 6:**
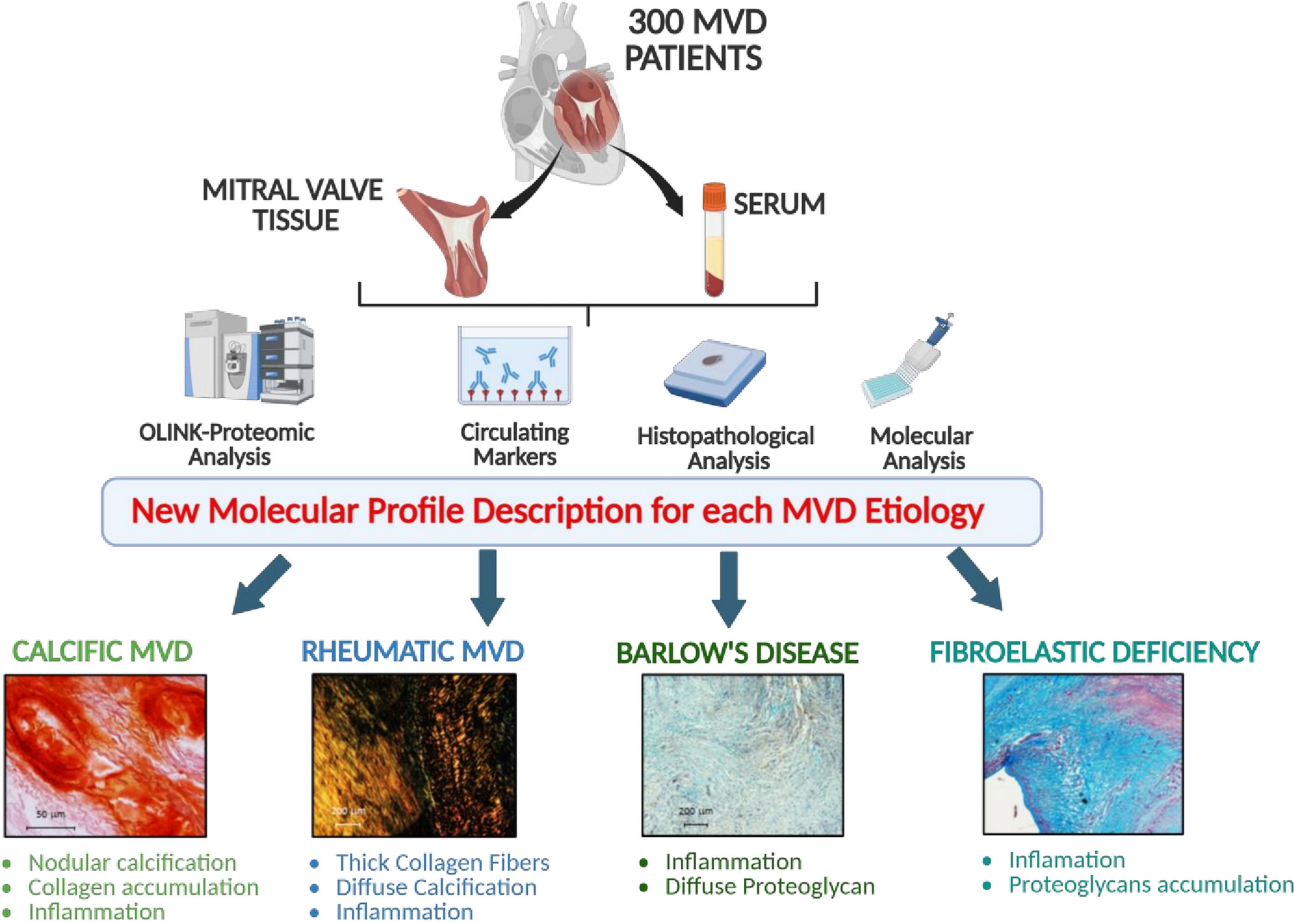
Circulating, molecular, and histopathological analysis for mitral valve disease (MVD) etiologies classification. 300 mitral valve patients (tissues and serum) were analyzed by OLINK-Proteomic, circulating markers, histopathological and molecular analyses. The present work demonstrated the presence of nodular calcification, collagen accumulation and inflammation in the calcific MVD; thick collagen fibers, diffuse calcification and inflammation in the rheumatic MVD; inflammation and diffuse proteoglycans in the Barlow´s disease; and inflammation ad proteoglycan accumulation in the fibroelastic deficiency.

In summary, in the present work, we demonstrate significant differences among the etiologic subgroups that imply different pathophysiological mechanisms in the development of MVD (Figure 6). However, longitudinal research to assess the role in disease evolution and therapeutic decision-making is needed. The knowledge of the differences between the MVD disease groups is essential to identify new specific therapeutic targets for each specific MVD. Our study reinforces the idea of the varied pathophysiology of MVD, and all types of MVD should be considered as active, complex, and unique processes. Thus, the term ‘degenerative’ MVD, although widespread, is misleading and should be carefully employed.

### Study limitations

The main limitation of our study is its descriptive and exploratory nature. Several potential mechanisms were found that need deeper analysis in further studies. Besides, we harvested the resected leaflets during MV replacement surgery, consequently in the final stage of the disease. For this reason, we are not able to measure markers in early-stage disease. Hence, it is difficult to discern whether the observed molecular and histological alterations in each subtype are causing the valvular dysfunction or are derived from a maladaptive response to previous injury instead. Nevertheless, our data informs how differently the pathology evolves in each form of MVD with potential clinical applications aiming to delay the damage within the MV. MV repair cases from our study were excluded because we do not have access to excised valve tissue from these patients, which is essential for analyzing the full panel of tissue markers. Finally, although we identified several molecular pathways that may be differentially implicated in certain etiologies, specific pharmacologic treatments are currently lacking, apart from TNF-α inhibitors whose possible role in RHVD might be worth exploring.

## Conclusions

To date, this is the largest comparative study analyzing the circulating, histological, and molecular alterations in MV from different primary chronic MV etiologies. Our study thoroughly reveals etiological-related differences with distinctive anatomical affectation and activation of pathophysiological mechanisms in the development of MVD.

## Supplementary material

online supplementary material 1.

## Data Availability

The datasets used and/or analyzed during the current study are available from the corresponding author on reasonable request.

## Abbreviations

AXL: tyrosine-protein kinase receptor UFO
BD: Barlow’s disease
CMVD: calcific mitral valve disease
ECM: extracellular matrix
FED: fibroelastic deficiency
GDF15: growth differentiation factor 15
GP6: glycoprotein
MMP: matrix metalloproteinase
MVD: mitral valve disease
OPN: osteopontin
PGLYRP1: peptidoglycan recognition protein
RHVD: rheumatic heart valve disease
TFF3: trefoil factor 3
TIMP: tissue inhibitors of metalloproteinase
