# Supplementary material for: Serum proteomics mirrors the histopathological changes underlying different etiologies of primary mitral valve disease
Source: Biosci Rep. 2026 Jan 14;46(1):BSR20253900. doi: 10.1042/BSR20253900 (PMC12863031; doi:10.1042/BSR20253900)
Supplement: online supplementary material 1. [file bsr-46-1-BSR20253900-s001.pdf]

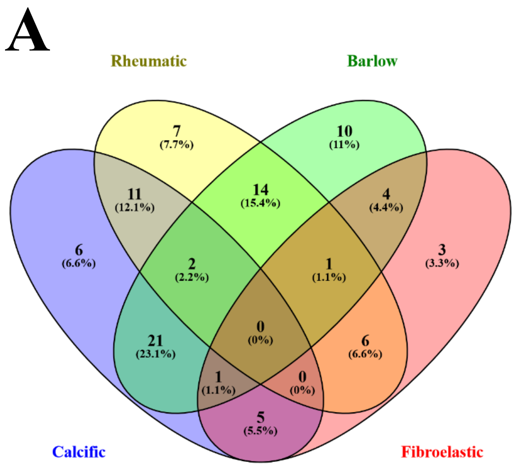

B

Etiology	Targets exclusively up-regulated	Targets significantly up-regulated
Calcific	JAM-A, CYSD, AXL, MCP-1, CPB1, GP6*	COL1A1, PECAM-1, u-PAR, RETN, IL-18BP, TLT-2, CHIT, OPN, GP6
Rheumatic	IGFBP-1, PGLYRP1, CXCL16, IL2-RA*, ST2*, GDF15, MB	PRTN3, TFF3, OPN, TRM, IL2-RA, ST2
Barlow	AZU1, PON3, TNSF13B, AP-N, CHI3L1, DLK-1*, IL-17R1. ICAM-2*, CD163, uPA*	PSP-D, TFF3, ALCAM, CCL15, RETN, IL-18BP, TLT-2, CHIT, ITGB-2, SHPS-1, TNFRSF10C, DLK-1, ICAM-2, uPA
Fibroelastic defficiency	EGFR*, PI3*, Ep-CAM	EGFR, PI3

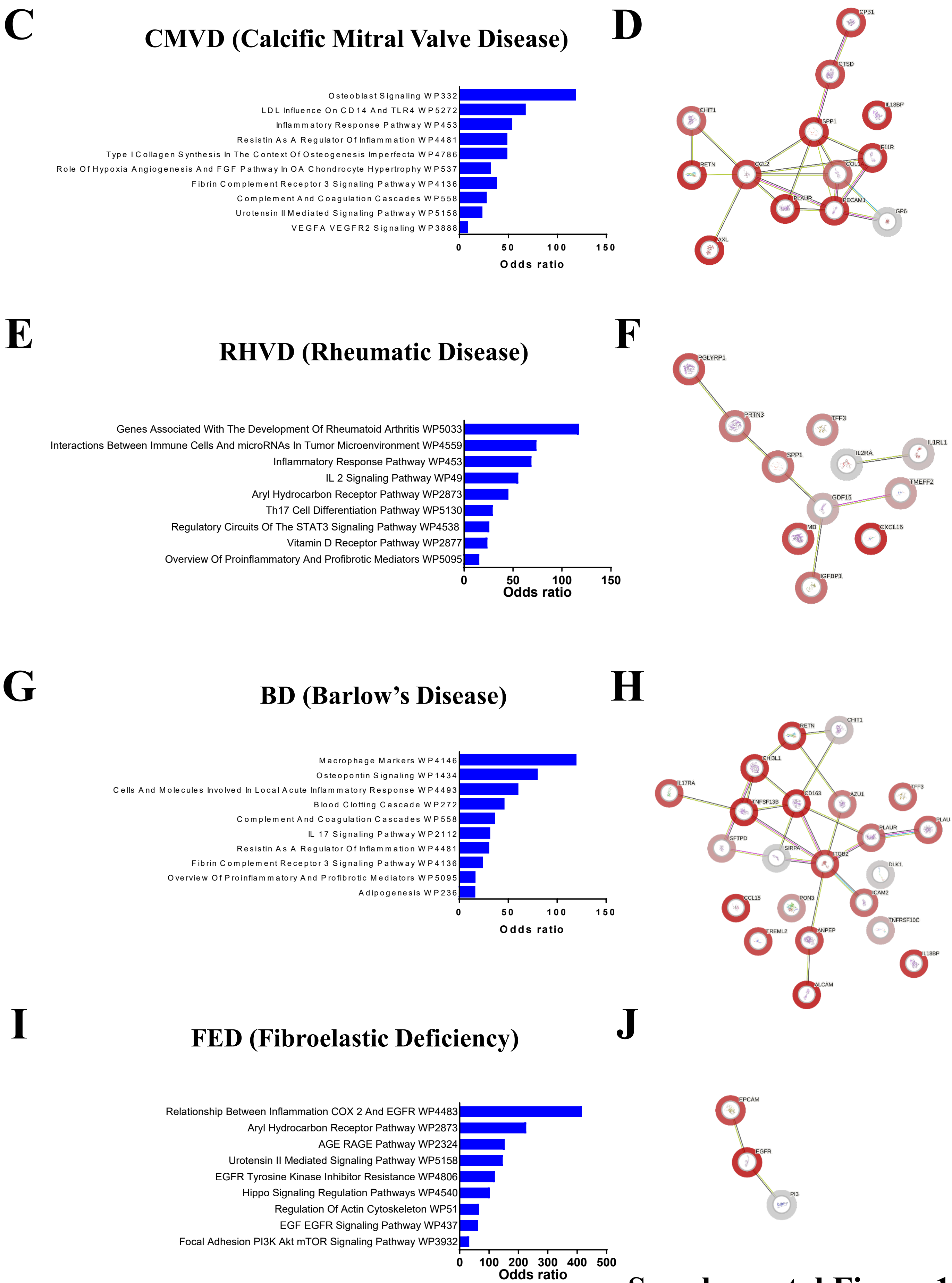

Supplemental Figure 1

Supplemental Figure 1. A, Venn diagram of the targets found up-regulated by OLINK in each MVD etiology. **B**, table summarizing these targets significantly up-regulated as well as these exclusively up-regulated, significantly (*) or tendentiously, in each etiology. Interactome and pathway enrichment in CMDV (**C-D**), RHVD (**E-F**), BD (**G-H**) and FED (**I-J**). N= 300 MVD valves were analyzed (N=81 for CMVD, N=114 for RHVD, N=70 for BD, N=35 for FED). MANOVA and Levene tests were performed. BD, Barlow's disease; CMVD, calcific degenerative mitral valve disease; FED, fibroelastic deficiency; RHVD, mitral valve disease. ** $p < 0.01$; *** $p < 0.001$; **** $p < 0.0001$.

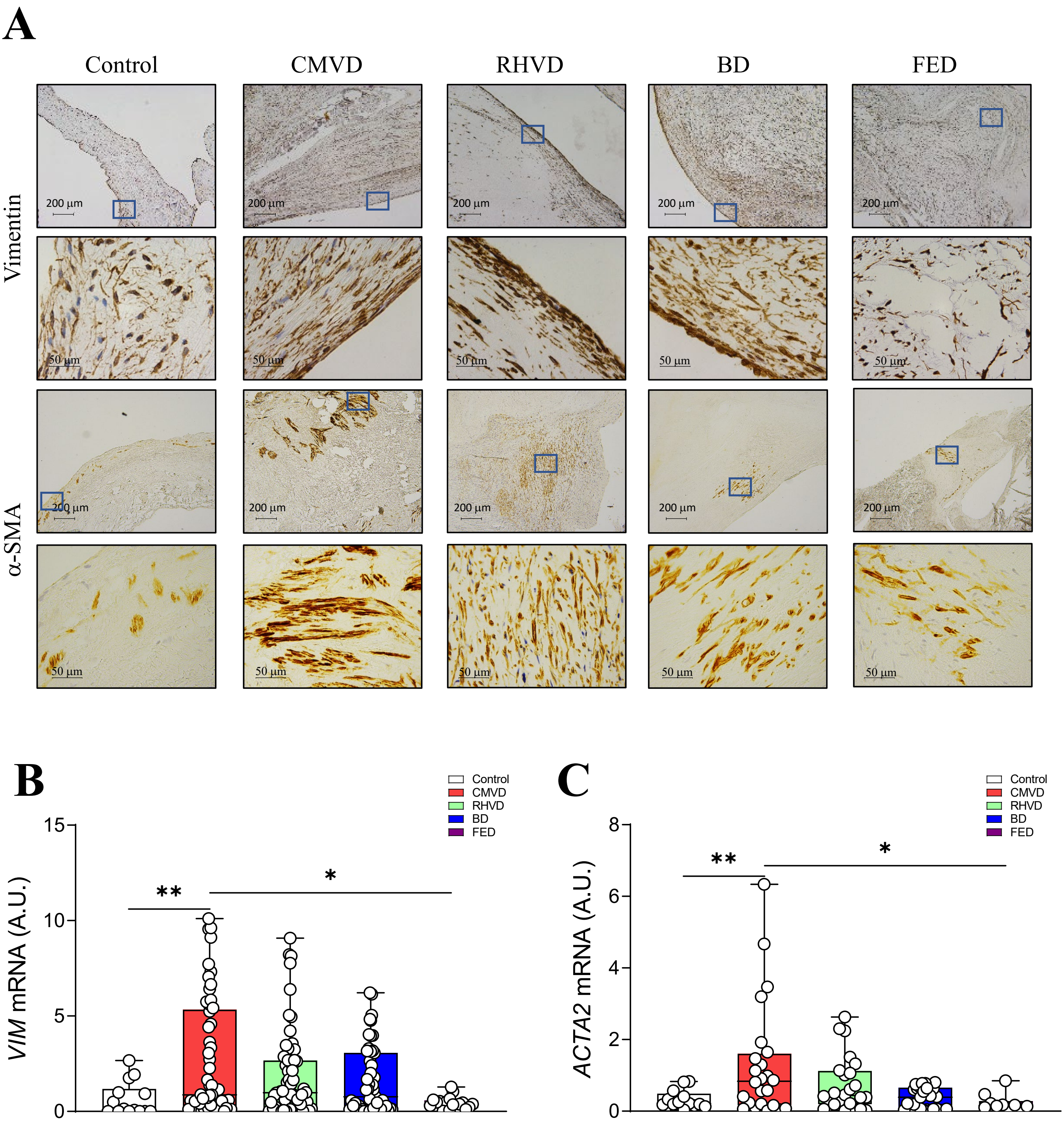

Supplemental Figure 2

Supplemental Figure 2. MV tissue homogenates of Control, CMVD, RHVD, BD and FED groups were assayed. **A.** Immunohistological characterization of VIC activation markers (Vimentin and α-SMA) at low (50X) or high (400X) magnification (top and bottom panels, respectively). **B.** Quantification of VIC activation markers (Vimentin and α-SMA) measured by PCR. N= 300 MVD valves were analyzed (N=81 for CMVD, N=114 for RHVD, N=70 for BD, N=35 for FED). One-way ANOVA or Kruskal Wallis test followed by Dunnett's or Mann-Whitney U test were performed. BD, Barlow's disease; CMVD, calcific degenerative mitral valve disease; FED, fibroelastic deficiency; RHVD, mitral valve disease. ** $p < 0.01$; *** $p < 0.001$; **** $p < 0.0001$.

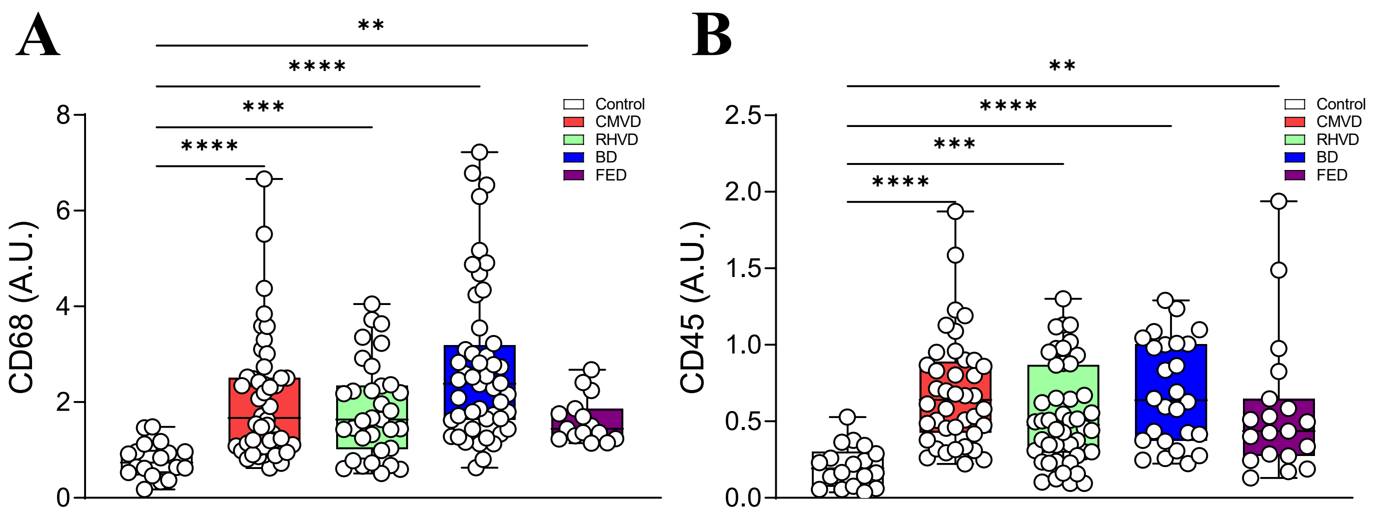

Supplemental Figure 3

Supplemental Figure 3. MV tissue homogenates of Control, CMVD, RHVD, BD and FED groups were assayed. **A-B** Quantification of macrophages infiltrates (CD68 and CD45) measured by PCR. N= 300 MVD valves were analyzed (N=10 for CMVD, N=10 for RHVD, N=10 for BD, N=10 for FED). At least 5 fields per MV were quantified. One-way ANOVA or Kruskal Wallis test followed by Dunnett's or Mann-Whitney U test were performed. BD, Barlow's disease; CMVD, calcific degenerative mitral valve disease; FED, fibroelastic deficiency; RHVD, mitral valve disease. ** $p < 0.01$; *** $p < 0.001$; **** $p < 0.0001$.

SUPPLEMENTAL MATERIAL

Supplemental Table 1. **Echocardiographic parameters.**

	**Calcific MVD**	**Rheumatic MVD**	**Barlow's disease**	**Fibroelastic deficiency**	***P* value**
LVEF, %	57.1 ± 12.6	61.1 ± 8.6	59.8 ± 10.8	60.1 ± 10.0	0.075
LVEF <60%	39 (48.1)	42 (36.8)	25 (35.7)	10 (28.6)	0.172
LVEF <50%	22 (27.2)	12 (10.5)	12 (17.1)	6 (17.1)	0.027
LVEF <35%	2 (2.5)	1 (0.9)	2 (2.9)	1 (2.9)	0.749
RV dysfunction	12 (14.8)	14 (12.3)	7 (10.0)	4 (11.4)	0.852
LV EDD, mm	53.0 ± 7.2	50.9 ± 6.2	59.7 ± 6.0	57.8 ± 8.2	<0.001
Indexed LV EDD, mm/m^2	30.2 ± 5.3	30.1 ± 3.5	32.8 ± 3.7	31.8 ± 4.2	<0.001
LV ESD, mm	36.2 ± 7.8	33.2 ± 5.2	40.7 ± 8.0	38.7 ± 8.3	<0.001
Indexed LV ESD, ml/m^2	20.9 ± 4.5	19.6 ± 3.1	23.3 ± 7.4	21.1 ± 4.3	<0.001
LV EDV, ml	126.8 ± 45.5	119.6 ± 48.3	168.3 ± 50.6	163.5 ± 57.1	0.008
Indexed LV EDV, ml/m^2	71.8 ± 26.5	65.4 ± 26.8	90.3 ± 25.2	90.6 ± 27.7	0.012
LV ESV, ml	66.4 ± 36.3	53.2 ± 27.3	76.1 ± 27.6	72.8 ± 21.7	0.099
Indexed LV ESV, ml/m^2	38.3 ± 22.1	28.9 ± 14.7	40.9 ± 14.5	40.0 ± 9.4	0.101
SPAP, mmHg	47.3 ± 11.4	49.4 ± 14.8	46.3 ± 12.4	48.3 ± 9.5	0.459

Data are presented as n (percentage) and mean ± standard deviation. LVEF, left ventricular ejection fraction; RV, right ventricle; LV, left ventricle EDD, end-diastolic diameter; ESD, end-systolic diameter; EDV, end-diastolic volume; ESV, end-systolic volume; SPAP, systolic pulmonary artery pressure.

Supplemental Table 2: Clinical characteristics including associated heart valve disease and surgery performed of the patients of the cohort depending on MVD etiologic subtype.

		Calcific MVD	Rheumatic MVD	Barlow's disease	Fibroelastic deficiency	*P* value
N (%)		81 (27)	114 (38)	70 (23)	35 (12)	
Age at surgery (years, mean ± SD)		74.7 ± 6.0	68.1 ± 8.4	67.0 ± 10.5	73.7 ± 8.6	<0.001
Male sex		31 (38.3)	26 (22.8)	45 (64.3)	26 (74.3)	<0.001
Hypertension		59 (72.8)	60 (52.6)	32 (45.7)	24 (65.6)	0.002
Hypercholesterolemia		47 (58.0)	63 (55.2)	31 (44.3)	16 (45.7)	0.275
Diabetes mellitus		24 (30.9)	16 (14.0)	5 (7.1)	3 (8.6)	0.001
Current or former smoker		21 (25.9)	43 (37.7)	23 (32.9)	13 (37.1)	0.322
Coronary heart disease		28 (34.6)	12 (10.5)	14 (20)	9 (25.7)	0.001
Atrial fibrillation		58 (71.6)	93 (81.6)	34 (48.6)	19 (54.3)	<0.001
Heart failure						
Prior admission		30 (37.0)	37 (32.5)	13 (18.6)	8 (22.9)	0.058
NYHA functional class	I	6 (7.4)	4 (3.5)	7 (10.0)	2 (5.7)	0.377
	II	31 (38.3)	51 (44.7)	35 (50.0)	18 (51.4)	
	III	43 (53.1)	59 (51.8)	27 (38.6)	14 (40.0)	
	IV	1 (1.2)	0 (0.0)	1 (1.4)	1 (2.9)	
Heart valve disease						
Mitral valve dysfunction	Mitral regurgitation	79 (97.5)	80 (70.2)	70 (100.0)	35 (100.0)	<0.001

(≥moderate)	Mitral stenosis	3 (3.7)	83 (72.8)	0 (0.0)	0 (0.0)	<0.001
Associated heart valve disease (≥moderate)	Aortic stenosis	26 (32.1)	22 (19.3)	0 (0.0)	5 (14.3)	<0.001
	Aortic regurgitation	27 (33.3)	26 (22.8)	10 (14.3)	9 (25.7)	0.048
	Tricuspid regurgitation	39 (48.1)	58 (50.9)	25 (35.7)	12 (34.3)	0.113
Heart surgery performed						
Isolated MV replacement (MVR)		16 (19.8)	38 (33.3)	45 (64.3)	19 (54.3)	<0.001
MVR + Aortic valve replacement (AVR)		33 (40.7)	26 (22.8)	6 (8.6)	9 (25.7)	
MVR + Tricuspid annuloplasty (TA)		27 (33.3)	40 (35.1)	16 (22.9)	6 (17.1)	
MVR + AVR + TA		5 (6.2)	10 (8.8)	3 (4.3)	1 (2.9)	
Coronary artery bypass graft		14 (17.3)	5 (4.4)	7 (10.0)	3 (8.6)	0.028

Data are presented as n (percentage) or mean ± standard deviation (SD), as appropriate. NYHA, New York Heart Association; MVR, mitral valve replacement; AVR, aortic valve replacement; TA, tricuspid annuloplasty; MVD, mitral valve disease.

Supplemental Table 3. Pharmacological treatment of the patients included at the time of surgery.

	Calcific MVD	Rheumatic MVD	Barlow's disease	Fibroelastic deficiency	*P* value
ACEi	25 (30.9)	28 (24.6)	27 (38.6)	13 (37.1)	0.195
ARB	26 (32.1)	18 (15.8)	10 (14.3)	10 (28.6)	0.013
Spironolactone	5 (6.2)	15 (13.2)	4 (5.7)	4 (11.4)	0.241
Eplerenone	3 (3.7)	2 (1.8)	0 (0.0)	2 (5.7)	0.234
Betablockers	55 (67.9)	80 (70.2)	38 (54.3)	16 (45.7)	0.018
Digoxin	18 (21.4)	50 (43.9)	14 (20.0)	3 (8.6)	<0.001
Statins	43 (53.1)	58 (50.9)	33 (47.1)	13 (37.1)	0.429
Acetylsalicylic acid	12 (14.8)	11 (9.6)	10 (14.3)	4 (11.4)	0.684
Acenocumarol/warfarin	49 (60.5)	92 (80.7)	27 (38.6)	18 (51.4)	<0.001
DOACs	3 (3.7)	0 (0.0)	1 (1.4)	1 (2.9)	0.230
Diuretics	69 (85.2)	104 (91.2)	50 (71.4)	29 (82.9)	0.005

Data are presented as n (percentage). ACEi, angiotensin converter enzyme inhibitor; ARB, angiotensin receptor blocker; DOACs, direct oral anticoagulants; MVD, mitral valve disease.

Supplemental Table 4. Results of laboratory test prior to surgery.

	Calcific MVD	Rheumatic MVD	Barlow's disease	Fibroelastic deficiency	*P* value
Hemoglobin (g/dl)	14.3 ± 10.1	13.5 ± 1.5	14.1 ± 1.4	13.5 ± 1.5	0.759
Creatinine (g/dl)	1.1 ± 0.8	0.9 ± 0.2	1.0 ± 0.7	1.0 ± 0.3	0.252
Creatinine clearance (ml/min/1.73m^2)	65.6 ± 28.1	69.4 ± 22.9	80.0 ± 29.4	72.0 ± 31.3	0.013
BNP (pg/ml)	378.9 ± 361.1	251.2 ± 190.7	250.2 ± 352.3	277.7 ±295.6	0.028
Total cholesterol (mg/dl)	177.3 ± 45.6	180.2 ±43.4	180.1 ± 41.4	176.2 ± 40.5	0.951
HDL-cholesterol (mg/dl)	47.3 ± 14.6	47.8 ± 20.7	46.7 ± 15.3	45.9 ± 14.8	0.957
LDL-cholesterol (mg/dl)	108.8 ± 37.2	112.8 ± 33.8	115.0 ± 31.5	109.9 ± 34.3	0.737
Triglycerides (mg/dl)	109.0 ± 50.4	111.6 ± 57.1	102.4 ± 52.6	100.4 ± 48.4	0.637

Data are presented as mean ± standard deviation. Creatinine clearance estimated by Cockroft-Gault formula. BNP, B type natriuretic peptide; HDL, high density lipoprotein; LDL, low density lipoprotein; MVD, mitral valve disease.
